# Cardiac Abnormalities in Feline Hyperthyroidism

**DOI:** 10.3390/vetsci12121115

**Published:** 2025-11-23

**Authors:** Birgit van Zuiden, Giorgia Santarelli, Sara Galac, Hans S. Kooistra, Viktor Szatmári

**Affiliations:** Clinical Sciences, Faculty of Veterinary Medicine, Utrecht University, Yalelaan 108, 3584 CM Utrecht, The Netherlands

**Keywords:** cats, echocardiography, dyspnea, heart failure, hypertrophic cardiomyopathy, NT-proBNP, pulmonary edema, pleural effusion

## Abstract

Hyperthyroidism is a common endocrine disorder in elderly cats, often leading to concurrent cardiac abnormalities. Understanding the development of these cardiac abnormalities and the effect of treatment is crucial for optimizing monitoring strategies and long-term management. Hyperthyroid cats frequently develop cardiomegaly, with left ventricular concentric and/or eccentric hypertrophy, and left atrial dilation. Cardiac abnormalities described in humans with multinodular toxic goiter may differ from those in cats, but, as in humans, these abnormalities are often reversible once thyroid hormone levels are normalized. Definitive treatment options for hyperthyroidism, such as radioiodine therapy and thyroidectomy, are the most successful at restoring the normal thyroid hormone levels. Medical therapy and iodine-restricted diets can also help normalize thyroid hormone levels, thereby aiding the reversal of cardiac abnormalities. However, cats with pre-existing cardiac diseases, like hypertrophic cardiomyopathy, may not show reversible cardiac changes due to these concurrent primary cardiac diseases. Cats with hyperthyroidism should routinely undergo echocardiographic evaluations to monitor for concurrent cardiac abnormalities, both before and after treatment. More importantly, every cat older than 6 years of age with echocardiographic or clinical signs of heart disease should be screened for hyperthyroidism. With appropriate treatment of hyperthyroidism, heart failure can be prevented.

## Simple Summary

Too much hormone production from the thyroid glands is a frequently occurring health issue in elderly cats. This condition is called feline hyperthyroidism. The most characteristic clinical signs of this disease include weight loss, hyperactivity, increased appetite, and thirst. Though the cause of the disease is unknown, effective treatment options are available. If hyperthyroidism is left untreated, it can have negative effects on several organ systems, including the heart. In some hyperthyroid cats, these cardiac changes do not cause any clinical signs, but in others, heart failure can occur, resulting in breathing difficulties and even death due to fluid accumulation in the lungs or in the chest. Feline hyperthyroidism is, in most cats, easy to diagnose with a routine blood test. Similarly to hyperthyroidism, genetic heart diseases occur commonly in elderly cats. With ultrasound examination of the heart, the cause of cardiac changes cannot be determined. But because cardiac changes caused by hyperthyroidism can be cured, it is of utmost importance that middle-aged to elderly cats with a suspected or proven heart disease be tested for the presence of hyperthyroidism.

## 1. Introduction

Feline hyperthyroidism is a frequently diagnosed endocrine disease in cats. In the majority of cases, the condition is caused by thyroid adenomatous hyperplasia or adenomas, which secrete excessive amounts of the thyroid hormones thyroxine (T4) and triiodothyronine (T3). Only in very few cases is hyperthyroidism caused by malignant thyroid carcinomas [1,2].

Hormone-producing thyroid adenomatous tissue in cats was rarely reported before 1979. Occasional pathological findings of thyroid nodules or masses had been identified in cats, but were seldom linked to clinical signs [1,3]. The first case was described in 1979 by Peterson and colleagues [4], and since then, the prevalence of feline hyperthyroidism has steadily increased to the point that it is now considered the most common endocrine disorder in middle-aged to elderly cats [1,2]. The reported prevalence may vary depending on the geographic area; it is estimated that around 10% of cats aged above 10 years in the United States have hyperthyroidism [2].

The precise cause for the development of hormone-producing thyroid adenomatous tissue remains unclear. Risk factors considered to possibly play a role include (1) nutritional imbalances in the diet, which cause metabolic thyroid dysfunction; and (2) environmental thyroid-disrupting substances, which interfere with thyroid hormone regulation [1]. Epidemiologic studies attempted to validate a variety of risk factors, but conflicting results leave their significance uncertain [2].

The elevated thyroid hormones stimulate various processes of metabolism, causing the body to go into a hyperactive state [5]. Classical clinical signs are weight loss, polyphagia, polyuria/polydipsia, vomiting, diarrhea/increased fecal volume, tachypnea (with occasional panting), tachycardia, increased activity, agitation, unkempt hair coat, and increased vocalization [1,2,5].

Cardiovascular comorbidities are frequently reported in feline hyperthyroidism [2]. The heart shows adaptations to the increased metabolism and hyperdynamic circulatory stage, such as left ventricular concentric hypertrophy (LVCH) and dilated cardiac chambers [6,7]. Auscultatory abnormalities such as murmurs, gallop sounds, and arrhythmias are commonly described, and congestive heart failure (CHF) has been reported in up to 10–15% of the hyperthyroid cats with cardiac abnormalities [6,7].

Echocardiography cannot determine with certainty whether LVCH in cats is due to hyperthyroidism, another endocrine disease such as hypersomatotropism, systemic arterial hypertension, an infiltrative myocardial disease, or hypertrophic cardiomyopathy (HCM) [6]. The latter is a primary myocardial disease that also shows a high prevalence (reported in up to 29% of older cats), and can occur simultaneously with hyperthyroidism [6,8].

Treatment of hyperthyroidism in cats includes palliative treatments with antithyroid medication or iodine-restricted diets, and definitive treatments such as radioiodine therapy or surgical thyroidectomy [9]. The effectiveness of different treatment options on the cardiac abnormalities caused by hyperthyroidism may vary. The aims of this literature review were to summarize the current knowledge on the cardiac effects of excess thyroid hormones in feline hyperthyroidism and the impact of different treatment options. Comparisons with human hyperthyroidism are made to gain insights into the applicability of human approaches in cats.

## 2. Results

### 2.1. Cardiac Effects of Thyroid Hormone Excess

Thyroid hormones play a crucial role in regulating metabolic processes. Because the functions of thyroid hormones are typically stimulatory, an excess of T4 and T3 leads to an overall increase in metabolic activity. This elevated metabolic state affects multiple organ systems, contributing to the characteristic clinical signs of hyperthyroidism. It also places a significant burden on the cardiovascular system, leading to functional and structural changes. These adaptive responses may eventually result in clinically relevant abnormalities [5].

The cardiac effects of hyperthyroidism are complex and can be associated with indirect or direct effects of thyroid hormone excess on the cardiac muscle. A precise understanding of the underlying pathophysiology has currently not been reached [10].

Indirectly, the increased metabolic activity leads to a greater need for tissue perfusion, which results in a high-output cardiac state. In this state, the afterload decreases secondary to an abnormally low systemic vascular resistance, and cardiac output increases. Renal compensatory mechanisms lead to volume overload, which leads to cardiac chamber dilation. Additionally, hyperthyroidism can alter the expression of genes regulating cardiomyocyte structure and function, contributing to myocardial hypertrophy. Furthermore, the upregulation of beta-1 adrenoceptors on cardiomyocyte cell membranes enhances inotropic, dromotropic, and chronotropic effects [8]. Thyroid hormones can also have a direct impact on the cardiac muscle, stimulating oxygen consumption and protein synthesis and therefore myocardial hypertrophy [7,11]. Hyperthyroidism has also been linked to inefficient utilization of high-energy phosphate compounds during cardiac contraction, which may further promote cardiac hypertrophy [7,10,12].

### 2.2. Cardiac Abnormalities in Feline Hyperthyroidism

Various studies have explored the presence of cardiac abnormalities in hyperthyroid cats [7,13,14,15,16,17,18,19,20,21,22,23].

On physical examination, soft systolic murmurs are often auscultated, with varying prevalence up to 90% [7,10,14,18,20,21,22,23]. Notably, murmurs are not always related to the presence of abnormal findings on echocardiography [22]. Other reported abnormalities include bounding pulses, tachycardia (>240 bpm), gallop sounds, and arrhythmias [7,14,18].

The most commonly reported abnormalities on electrocardiography are sinus tachycardia (heart rate ≥ 240 bpm) and tall R-waves (≥0.9 mV) [10,13,14,15,19]. Low frequency of atrial and ventricular arrhythmias, atrioventricular blocks, and intraventricular conduction abnormalities has also been identified [10,13,14,19].

On thoracic radiography, cardiomegaly has commonly been observed [14,15,20,22]; however, the methodology to determine this finding has not been systematically described, making comparison among studies difficult. Evidence of CHF has also been identified [14,19]. The prevalence of CHF possibly relates to the severity of hyperthyroidism, with a reduced incidence since earlier diagnosis occurs [18,21].

Several studies have investigated the echocardiographic findings that can be observed in cats with hyperthyroidism. The most commonly observed abnormalities include LVCH, left ventricular chamber dilation, left atrial enlargement, and left ventricular diastolic dysfunction [7,15,16,17,21,22]. However, echocardiography does not always identify changes, with up to 51% of hyperthyroid cats showing no remarkable abnormalities [20,21,22]. Such variation could possibly relate to the severity of the endocrine disease, with cardiac abnormalities more often observed in cats with higher T4 concentrations [21]. A dilated left ventricle with reduced systolic function, and therefore a dilated cardiomyopathy phenotype, has been described in eight cats with CHF [7,16]; raising the question whether such a finding could be more common in advanced stages of the disease. Regarding LVCH, this has been observed focally, involving the interventricular septum or the left ventricular free wall, or as a more diffuse change [15,21]. It should be noted that not all the studies mentioned have used the commonly accepted criterion for LVCH diagnosis of an end-diastolic wall thickness ≥ 6 mm [6]; however, higher dimensions than in control healthy subjects have often been identified.

Systemic arterial hypertension could also be a cause of LVCH [6]. Systemic hypertension has been identified in hyperthyroid cats, but this was not always related to LVCH [21]. Due to the significant limitations of non-invasive blood pressure measurement in cats, the true prevalence of systemic arterial hypertension in hyperthyroid cats cannot be accurately established, similar to its influence on echocardiographic findings.

On gross cardiac pathology, LVCH has also been commonly observed [8,10]. In a study comparing cats with HCM and cats with hyperthyroidism, 47% of hyperthyroid cats had LVCH, and this was often less prominent than in cats with HCM. Furthermore, septal involvement was more common in cats with HCM, although not specific [8]. On histopathology, myocardial degeneration and cardiomyocyte hypertrophy were seen in both groups. However, myocardial cell disarray was significantly more prevalent in the HCM group [8]. Another study reported similar findings, with only a small percentage of cats with LVCH due to hyperthyroidism showing marked disorganization of cardiomyocytes [10].

Cardiac biomarkers have also been shown to be abnormal in cats with hyperthyroidism. Detectable serum cardiac troponin I (cTnI) levels were present in 11/23 hyperthyroid cats prior to radioactive iodine treatment. Interestingly, raised serum cTnI concentrations corresponded to higher T4 levels [24]. Elevated serum cTnI levels indicate myocardial damage. In another study, also including 23 hyperthyroid cats, both plasma N-terminal pro B-type natriuretic peptide (NT-proBNP) and cTNI concentrations were higher than in healthy cats, but not significantly different from those of cats with HCM, therefore proving nonspecific for any particular condition [25]. A modest but significant effect of hyperthyroidism on NT-proBNP concentration was confirmed by another study including 61 hyperthyroid cats, reinforcing that care is needed when interpreting this biomarker in cats [26]. Elevated plasma NT-proBNP concentrations indicate increased ventricular wall stretch, where dilatation/eccentric hypertrophy produces a more pronounced increase than concentric hypertrophy.

### 2.3. Comparison Between Feline and Human Hyperthyroidism

Feline hyperthyroidism resembles clinically and histologically human toxic nodular goiter, also called Plummer’s disease. This condition is characterized by autonomous and thyroid-stimulating hormone (TSH)-independent activity of hyperfunctioning hyperplastic/adenomatous thyroid nodules releasing high concentrations of thyroid hormones [27,28,29]. The autonomy of toxic nodular goiter tissue has been demonstrated through experimental transplantation studies in nude mice. Both human and feline thyroid toxic nodular tissue continued to grow after transplantation without extrathyroidal humoral stimulating hormones [30,31]. Similar to its human counterpart, feline toxic nodular goiter is a progressive disease. The hyperplastic nodules increase in size over time, coalescing to adenomas and, in a small percentage of cases, even becoming malignant, especially when palliative treatment with thyreostatics is applied [32]. Another common risk factor for nodular goiter in both humans and cats is increasing age and exposure to environmental endocrine-disrupting compounds [28].

The main cardiac manifestations of human hyperthyroidism differ from those of feline hyperthyroidism. While LVCH is the most common finding in cats, a dilated cardiomyopathy phenotype is more frequently observed on echocardiography in humans [33,34,35,36,37,38]. In addition, pulmonary hypertension is a highly prevalent finding in human hyperthyroidism, which has been related to the high cardiac output [33,34,35,36,37,39]. In hyperthyroid cats, no evidence of pulmonary hypertension has been identified on echocardiography; however, this technique is known to have important limitations for this purpose [40].

Atrial fibrillation is the most common rhythm disturbance described in people with hyperthyroidism [35,36,37,39,41], and its presence is a major predictor of heart failure development [37]. Atrial fibrillation has not been commonly reported in cats with hyperthyroidism, which might reflect the large difference in the absolute size of the atria between the two species. While cats must have a severe atrial dilatation to develop atrial fibrillation, humans can develop this arrhythmia even with normal-sized atria.

### 2.4. Treatment Options for Feline Hyperthyroidism

Hyperthyroidism is a progressive disease, and if left untreated, it can lead to more severe clinical signs and a higher risk of comorbidities. Effective management is essential to improve the quality of life and long-term prognosis. The primary goals of treatment are to restore euthyroidism, avoid hypothyroidism, and minimize treatment-associated side effects [42].

There are four main therapeutic approaches to treat feline hyperthyroidism: radioiodine therapy, surgical thyroidectomy, antithyroid medication, and dietary management with iodine-restricted food. Each treatment has its own advantages and disadvantages. Eventually, the treatment choice is determined by multiple factors, depending on, for example, the cat’s age, existing comorbidities, financial considerations, the pet owner’s preferences, treatment accessibility, and the clinician’s recommendation and expertise. Most veterinarians advocate for definitive treatment with radioiodine or thyroidectomy, particularly in younger cats that are otherwise healthy. However, for geriatric cats, those with concurrent non-thyroidal conditions like renal or cardiac disease, or cases where owners opt against definitive therapy, long-term management with antithyroid medications or an iodine-restricted diet remains a viable alternative [2].

#### 2.4.1. Radioiodine Therapy

Radioiodine (I-131) therapy is a simple and effective treatment for feline hyperthyroidism. The radioactive iodine isotope can be administered either intravenously, subcutaneously, or orally, and it is selectively taken up by hyperactive thyroid tissue, emitting beta particles and gamma radiation that destroys abnormal thyroid cells, resulting in a definite cure of the hyperthyroidism [43]. A positive aspect of radioactive iodine administration is that it spares adjacent parathyroid tissue and has no systemic effects. Furthermore, most cases require only a single administration of radioactive iodine. Studies have shown a significant decrease in T4 levels within 30 days post-treatment, with over 90% of cats achieving euthyroidism [20,43].

Cats with severe hyperthyroidism often struggle with persistent disease and difficulties in achieving stable management. This group has a notably higher likelihood of developing cardiac complications, which can influence their eligibility for definitive treatments like radioiodine therapy. To minimize these risks, radioiodine therapy is preferred whenever possible before hyperthyroidism becomes severe, as earlier intervention may reduce comorbidities and lower the required treatment dose [21].

#### 2.4.2. Thyroidectomy

Surgical removal of the thyroid gland(s), i.e., thyroidectomy, is another curative option for cats with hyperthyroidism, with a success rate of more than 90% [44]. However, thyroidectomy carries surgical risks and anesthetic complications, especially in cats with a concurrent cardiac disease [45]. Scintigraphy is advised in cats prior to thyroidectomy, to evaluate uni- or bilateral involvement of thyroid disease and the presence of ectopic thyroid tissue [46]. Cats undergoing bilateral thyroidectomy require lifelong supplementation with l-thyroxine. Failure to preserve parathyroid tissue can result in life-threatening hypocalcemia and the need for medical management of hypoparathyroidism [44]. To reduce the risk of iatrogenic hypoparathyroidism, bilateral thyroidectomy can be staged, with a 4-week interval between surgeries [47].

#### 2.4.3. Medical Management

Antithyroid medication is the most commonly used treatment option for feline hyperthyroidism. It can be used lifelong, but also for a short term to achieve euthyroidism before surgery or radioactive iodine treatment. Two licensed antithyroid drugs are available in Europe: methimazole and its pro-drug carbimazole [9,45,48].

Antithyroid medication inhibits thyroid hormone synthesis and is available in oral and transdermal formulations. Because transdermal formulations have lower bioavailability and slower onset of action when compared to oral formulations, a higher dose of transdermal gels may be required to resolve the clinical signs of hyperthyroidism [49].

Regular monitoring of thyroid and renal function is essential during treatment with thyreostatics to ensure the efficacy of treatment and to avoid iatrogenic hypothyroidism, which can contribute to azotemia and in turn decreased survival [50]. While medical therapy is effective in reducing thyroid hormone levels, methimazole therapy is associated with a shorter median survival time compared to radioiodine therapy or thyroidectomy [20,51]. This could be related to owner compliance, but development of resistance to antithyroid drug therapy has also been advocated [20]. In fact, thyreostatic therapy does not affect the proliferation of hyperplastic/adenomatous thyroid tissue; therefore, the progression of the number, size, and volume of thyroid nodules can occur over time. Furthermore, in some cases, a benign thyroid tumor may transform into a malignant one, which is also less responsive to thyreostatics [32]. Owners should also be informed about the potential risks to their own health when handling antithyroid medications: these must be handled with care (e.g., by wearing gloves) to prevent accidental absorption and inadvertent self-medication [45].

#### 2.4.4. Dietary Management

An alternative non-invasive treatment for feline hyperthyroidism involves chronic feeding of an iodine-restricted prescription diet. This diet limits iodine intake, thereby reducing thyroid hormone production [2]. Studies have demonstrated its effectiveness in lowering T4 levels, although some cats may remain mildly hyperthyroid [23,52]. Compared to methimazole, dietary therapy has not been associated with an increase in serum creatinine levels, suggesting a potential protective effect on renal function or inferior effectiveness despite normal T4 levels. Importantly, compliance could be a challenge, as cats can refuse the diet due to poor palatability. Moreover, dietary management does not address the underlying thyroid pathology and may not provide long-term disease control [23].

### 2.5. The Effects of Hyperthyroidism Treatment on Cardiac Abnormalities in Cats

Since treatment can restore normal thyroid hormone concentrations, an important question is whether it also contributes to the resolution of cardiac abnormalities associated with feline hyperthyroidism. Follow-up echocardiographic examinations after treatment for hyperthyroidism can help determine whether the abnormalities fully resolve (i.e., reverse remodeling) after achieving euthyroidism.

The studies that have examined the effects of different treatment options for hyperthyroidism on cardiac abnormalities suggest that many of these changes improve or resolve upon restoration of a euthyroid state, after a variable period of time [7,13,15,53,54]. If cardiac changes persist despite good control of the hyperthyroidism, it could mean that either a longer time is needed for reverse remodeling to take place, a permanent cardiac damage has occurred, or the cat has an echocardiographically indistinguishable, concurrent primary cardiac disease [7].

Auscultatory abnormalities have been shown to decrease in frequency with effective treatment, including a decrease in heart rate [13,24]. A murmur was still present in four out of 10 cats originally presenting with a murmur six months after radioiodine therapy in a study. In the same study, a gallop rhythm was detected in 4/23 cats before treatment and only in one cat following treatment [24].

In a study including 45 hyperthyroid cats examined with electrocardiography, 17 of which were reevaluated 6 months after thyroidectomy when they were euthyroid, a significant decrease in heart rate and R-wave amplitude, as well as Q-T interval prolongation, was observed. Furthermore, atrial and ventricular arrhythmias resolved [13].

In another study in which hyperthyroid cats were treated with either thyroidectomy or radioiodine, echocardiographic abnormalities improved or resolved in 16/24 cats, as demonstrated by repeat examination 4 to 21 months after euthyroidism was achieved. A significant reduction in LVCH was observed, and atrial size was also reduced, as well as normalization of contractility indices and mean resting heart rate. However, 8/24 cats showed stable or progressive cardiac disease [7].

Another study that monitored seven cats with electrocardiographic, echocardiographic, or radiographic evidence of cardiomegaly showed that cardiac size gradually decreased within 18 months after thyroidectomy in most cats [15]. Improvements were also observed while cats were receiving antithyroid medication prior to surgery. More specifically, all cats showed a decrease in R-wave amplitude after definitive treatment, and 3/4 cats showed a measurable decrease in radiographic heart size. On echocardiography, 2/4 cats showed reduced left ventricular wall thickness, one showed no change, and another cat exhibited progressive LVCH. Interestingly, in one case where hyperthyroidism reoccurred 19 months after thyroidectomy, cardiomegaly reappeared, emphasizing the effects of excess thyroid hormones on cardiac morphology [15].

A larger echocardiographic study followed 91 hyperthyroid cats treated with orally administered radioiodine for two to three months [53]. The study observed significant improvement of hypertrophic changes in the left ventricular wall and interventricular septum. However, left ventricular chamber diameters remained outside the reference range (i.e., persistence of eccentric hypertrophy) in approximately 20% of cats at two months post-treatment. Moreover, a subset of cats developed new cardiac abnormalities post-treatment [53].

In a cohort study involving 231 hyperthyroid cats treated with radioactive iodine, 86% of the cats had cardiac-related abnormalities before treatment, including tachycardia, arrhythmias, or an HCM-phenotype [54]. At the final follow-up examination, with a median of 25 months post-treatment, only 7/57 cats (12%) had cardiac abnormalities. Furthermore, cardiac-related problems at death were reported in 4% of cases. Although the exact distribution of specific cardiac abnormalities and their diagnostic criteria were not reported, the findings suggest that a substantial number of cardiac-related abnormalities were resolved in this long-term study after radioactive iodine treatment [54].

Other studies including cats treated with radioiodine therapy showed a reduction in the concentrations of cTnI and NT-proBNP within 2–3 months following treatment [24,25]. In one of these studies, the reduction in biomarker concentrations was associated with a reduction in left ventricular wall thickness on echocardiography [25].

Another study, including 140 cats referred for radioiodine therapy, offered further insights into the cardiac effects of medical (95%) and dietary (5%) management of feline hyperthyroidism [21]. More than 50% of these cats were still biochemically hyperthyroid at presentation despite treatment. Furthermore, 45 cats had moved up at least one severity group (from mildly to moderately, or from moderately to severe hyperthyroid) prior to referral, based on T4 concentration. Many cats presented with cardiac abnormalities: next to a high prevalence of systolic murmurs (up to 90% in cats with severe hyperthyroidism), 51% had echocardiographic changes, and 1.4% had CHF [21]. This study highlights that non-definitive therapies may not always be ideal to manage hyperthyroidism, and therefore also not the secondary cardiac abnormalities.

### 2.6. The Effects of Hyperthyroidism Treatment on Cardiac Abnormalities in Humans

The same treatment options described for feline hyperthyroidism are available for humans with hyperthyroidism, with the exception that no study has reported the use of iodine-restricted diets in people. A frequently chosen treatment, alongside radioiodine therapy, is thyroidectomy [55]. Thyroidectomy is preceded by normalization of T4 levels using antithyroid medication. In human patients, methimazole or carbimazole are commonly prescribed, although propylthiouracil (in combination with carbimazole) and benzylthiouracil are also utilized [33,35,36,37]. In addition, due to increased sympathetic activity, beta-blockers are often administered alongside antithyroid medication, and in cases where cardiac dysfunction is diagnosed, additional cardiac medication is recommended [33,34,36,37].

Concerning the effects of hyperthyroidism treatment on secondary cardiac disease, the reported results vary, but improvement or resolution of cardiac changes are often described after euthyroidism is achieved with different therapeutic options [33,34,36,37,39]. However, evidence suggests that persisting cardiovascular abnormalities after effective treatment may contribute to significant cardiovascular morbidity in some cases [33]. A few studies showing the variable effects of different types of treatments are reported below.

A study investigating 41 hyperthyroid human patients with cardiac dysfunction undergoing thyroidectomy found that preoperative treatment with antithyroid medication, leading to a euthyroid state, already significantly improved cardiac parameters [37]. Three months post-surgery, all patients included showed complete reversal of atrial fibrillation and pulmonary hypertension. Furthermore, improvement or full recovery of echocardiographic abnormalities occurred in most cases with a dilated cardiomyopathy phenotype. Serum NT-proBNP levels showed a strong correlation with the decrease in serum thyroid hormone levels and improvement of echocardiographic parameters [36]. A follow-up study by the same authors evaluated two groups of patients up to six months post-surgery: one received only antithyroid medication, and another underwent thyroidectomy after stabilization with medical therapy. The results were consistent with the earlier study, showing significant cardiac amelioration in both groups, though the subjects who had thyroidectomy exhibited more pronounced improvement [37].

A study evaluating cardiac function in 39 hyperthyroid patients receiving medical therapy re-examined the included subjects once thyroid hormone levels had normalized. This study found a significant reduction in pulmonary arterial systolic pressure after treatment, with values becoming similar to those of euthyroid control subjects [39]. Another study included 32 hyperthyroid patients without pre-existing structural heart disease, with and without pulmonary hypertension. Post-treatment with methimazole or radioiodine therapy, significant reductions were observed in cardiac parameters in both groups, reflecting improvements in both right and left ventricular systolic and diastolic function [34].

Another study investigated 393 hyperthyroid patients compared with the same number of age- and gender-matched euthyroid control subjects [33]. Hyperthyroid patients were re-evaluated after antithyroid therapy with medication or radioiodine. Repeat assessment included attainment of cardiovascular history and examination, 12-lead electrocardiography, and 24 h Holter monitoring at approximately 6 and 9 months after initial evaluation. After excluding all subjects with known cardiovascular disease, cardiovascular symptoms, abnormal hemodynamics, and cardiac dysrhythmias were more prevalent among hyperthyroid patients at recruitment compared with control subjects. Such higher prevalence persisted also when the subjects were subclinically hyperthyroid or rendered euthyroid after treatment. However, in treated hyperthyroid patients without pre-existing cardiovascular disease, atrial fibrillation often resolved after achieving a euthyroid state [33].

## 3. Conclusions

Secondary heart disease is a common comorbidity of feline hyperthyroidism. The elevated concentrations of thyroid hormones increase tissue metabolism, causing a high-output cardiac state, to which the heart adapts and remodels. The main cardiac abnormalities described in cats with feline hyperthyroidism are left ventricular concentric and/or eccentric hypertrophy and left atrial dilation. Since CHF can occur in some cases, and its prevalence seems to relate to the severity of hyperthyroidism, early diagnosis and treatment of the hyperthyroidism are crucial, while parallel assessment of the cardiac status is recommended. For an early diagnosis of hyperthyroidism, thyroid hormone levels should be checked routinely in middle-aged to older cats, even in the absence of palpable goiter, for example, at yearly health checks, as clinical signs are not always evident.

Clinical and pathological aspects of feline hyperthyroidism resemble human toxic nodular goiter; however, there appears to be different compensatory mechanisms for high cardiac output in humans and cats. In humans, concentric hypertrophy is less frequently reported, while chamber dilation and pulmonary hypertension are more prominent. Additionally, atrial fibrillation is commonly reported in humans with hyperthyroidism but not in cats.

Various studies in cats and humans show that cardiac abnormalities observed with hyperthyroidism are at least partially reversible in the absence of pre-existing primary cardiac disease, with definitive therapies seeming more effective in the management of both the endocrine and the secondary heart disease.

Cardiac abnormalities in hyperthyroid cats and cats with primary (hypertrophic) cardiomyopathies are similar even in post-mortem examinations, complicating the achievement of a definitive diagnosis with echocardiography. Therefore, hyperthyroidism should always be looked for in cats with echocardiographic HCM phenotype (i.e., LVCH), left ventricular eccentric hypertrophy, or CHF, and treated appropriately if present. Subsequently, cardiac changes should be followed up to assess their progression. As opposed to changes due to primary cardiomyopathies, reverse remodeling of cardiac abnormalities caused by hyperthyroidism can occur after successful treatment. However, it should be taken into account that this process might take several months, and that the longer a cat has had hyperthyroidism, the more severe the cardiac abnormalities are likely to become, with less chance for full recovery. Furthermore, if euthyroidism is not achieved, which is more likely with medical or dietary therapies, the effects of thyroid hormone excess on the heart will also continue, potentially leading to a poorer overall prognosis.

## Data Availability

No new data were created or analyzed in this study.

## Abbreviations

The following abbreviations are used in this manuscript:

ACVIM	American College of Veterinary Internal Medicine
CHF	congestive heart failure
cTnI	cardiac troponin i
HCM	hypertrophic cardiomyopathy
LVCH	left ventricular concentric hypertrophy
NT-proBNP	N-terminal pro B-type natriuretic peptide
T3	triiodothyronine
T4	thyroxine
TSH	thyroid-stimulating hormone
